# First Insights into the Genome of the Cr(VI)-Reducing Bacterium *Clostridium chromiireducens* DSM 23318

**DOI:** 10.1128/genomeA.00420-17

**Published:** 2017-06-01

**Authors:** Anja Poehlein, Nils Höche, Alexander Mehr, Rolf Daniel

**Affiliations:** a Genomic and Applied Microbiology and Göttingen Genomics Laboratory, Institute of Microbiology and Genetics, Georg-August University of Göttingen, Göttingen, Germany; b Members of the Applied Bioinformatics in Microbiology Course of the Microbiology and Biochemistry MSc/PhD Program, Georg-August University of Göttingen, Göttingen, Germany

## Abstract

*Clostridium chromiireducens* is an obligate, anaerobic, Gram-positive, rod-shaped, and spore-forming bacterium that is able to reduce Cr(VI). The draft genome consists of one chromosome (5,448 Mb) and contains 4,773 predicted protein-encoding genes.

The obligate, anaerobic, spore-forming bacterium *Clostridium chromiireducens* is Gram-positive and rod-shaped. *C. chromiireducens* metabolizes glucose by mixed acid fermentation and is able to reduce Cr(VI) in low concentrations (1). Comparative 16S rRNA gene-based analysis revealed that *C. chromiireducens* belongs to cluster I of the genus *Clostridium*, with *C. beijerinckii* and *C. roseum* as closest relatives (1). *C. chromiireducens* was isolated from chromium-contaminated soil in a Superfund site in the upper peninsula of Michigan (1).

Chromosomal DNA of *C. chromiireducens* was isolated using the MasterPure complete DNA purification kit as recommended by the manufacturer (Epicentre, Madison, WI, USA). The extracted DNA was employed to generate Illumina paired-end sequencing libraries according to the manufacturer’s protocol (Illumina, San Diego, CA, USA). The libraries were sequenced using a MiSeq instrument and MiSeq reagent kit version 3 as recommended by the manufacturer (Illumina). Sequencing resulted in 3,313,510 paired-end reads that were trimmed using Trimmomatic version 0.36 (2). Genome sequence assembly with SPAdes version 3.10.0 (3) yielded 188 contigs (>500 bp) and an average coverage of 114-fold. For validation of the assembly, QualiMap version 2.1 was used (4). The draft genome exhibited a size of 5.448 Mb and a G+C content of 30.12%. The software Prokka (5) was used for automatic gene prediction and annotation. The draft genome contained 16 rRNAs, 79 tRNAs, and 1 tmRNA. From 4,773 predicted protein-encoding genes, 3,630 have a predicted function and 1,143 were coding for hypothetical proteins.

The acidic fermentation products of *C. chromiireducens* are acetate, butyrate, formate, and lactate (1). Correspondingly, putative protein-encoding genes for acetate kinase, phosphotransacetylase, phosphate butyryltransferase, butyrate-acetoacetate CoA-transferase, butyrate kinase, formate acetyltransferase, and lactate dehydrogenase are present in the genome. *C. chromiireducens* utilizes maltose, mannose, and sucrose as substrates (1) and harbors genes encoding the maltose and mannose phosphotransferase system and the sucrose hydrolase. Additionally, 39 putative multidrug resistance genes have been detected in the draft genome sequence. Cr(VI) is a toxic compound leading to membrane damage (6). Cr(VI) resistance can be established via efflux transporters or reductases. It has been shown that *C. chromiireducens* reduces Cr(VI) to Cr(III) (1). Enzymatic chromate reduction is usually mediated by NAD(P)H-dependent flavin reductases (7). *C. chromiireducens* possesses a putative gene encoding NADPH-dependent flavin oxidoreductase (*frp*), which is a potential candidate for Cr(IV) reduction. An operon putatively encoding copper resistance consisting of genes encoding a copper-sensing transcriptional repressor, a copper-exporting P-type ATPase, and a copper chaperone was present in the genome. Another putative copper-facilitating operon was linked to a fluoride channel and a fluoride ion transporter.

## Accession number(s).

This whole-genome shotgun project has been deposited at DDBJ/EMBL/GenBank under the accession number MZGT00000000. The version described here is the first version, MZGT01000000.
